# Universal masking in hospitals in the COVID-19 era: Is it time to consider shielding?

**DOI:** 10.1017/ice.2020.179

**Published:** 2020-04-29

**Authors:** Sonali D. Advani, Becky A. Smith, Sarah S. Lewis, Deverick J. Anderson, Daniel J. Sexton

**Affiliations:** 1 Division of Infectious Diseases, Department of Medicine, Duke University School of Medicine, Durham, North Carolina; 2 Duke Center for Antimicrobial Stewardship and Infection Prevention, Durham, North Carolina

## Abstract

With concerns for presymptomatic transmission of COVID-19 and increasing burden of contact tracing and employee furloughs, several hospitals have supplemented pre-existing infection prevention measures with universal masking of all personnel in hospitals. Other hospitals are currently faced with the dilemma of whether or not to proceed with universal masking in a time of critical mask shortages. We summarize the rationale behind a universal masking policy in healthcare settings, important considerations before implementing such a policy and the challenges with universal masking. We also discusses proposed solutions such as universal face shields.

As the novel coronavirus (SARS-CoV-2) public health crisis escalates, several hospitals have supplemented pre-existing infection prevention measures, such as visitor restrictions and employee screening, with universal masking of all healthcare professionals (HCPs). A universal masking policy usually requires that all HCPs (clinical and nonclinical) wear some sort of face mask while on hospital premises. These new policies also continue pre-existing policies requiring the use of N95 respirators (when available) when performing aerosol-generating procedures on patients with known or suspected SARS-CoV-2. In a nutshell, the rationale of implementing a universal masking policy in hospitals is to limit the transmission of SARS-CoV-2 from patients to HCP and from HCP to patients and/or to other HCPs. In the following sections, we summarize the rationale for universal masking in hospitals, important considerations before implementing this policy, and the challenges with universal masking, and we discuss proposed solutions such as universal face shields.

## Rationale for universal masking

Atypical presentations and presymptomatic transmission of SARS-CoV-2 have now been shown to cause clusters of COVID-19 in community settings, nursing homes, cruise ships, and returning travelers.^1–3^ For example, approximately half of the residents in a skilled nursing facility in Washington who tested positive as a result of an exposure investigation were not symptomatic on the day of testing.^1^ Of the 114 persons in a cohort of returning travelers who tested positive for SARS-CoV-2, 2 (1.8%) were asymptomatic on screening.^2^ Similarly, almost half of the 712 persons with a positive test result on the Diamond Princess cruise ship were asymptomatic at the time of testing.^3^ Most recently, an investigation of 7 clusters in Singapore provided further evidence that viral shedding can occur before symptom onset.^4^ This may result in transmission from presymptomatic HCPs to patients and other HCPs, although frequency of transmission from such individuals is an unresolved question. However, these exposure investigations usually occur after symptom onset, which increases the burden of contact tracing and the number of exposed HCPs placed on furlough. A surgical mask also provides a physical barrier between hands and mucus membranes of mouth and nose. An average person touches their face spontaneously ~23–26 times per hour. A mask serves as a constant reminder to reduce hand-to-face contact.

## Important considerations when implementing universal masking

An adequate supply of masks is an obvious prerequisite for implementing a universal masking policy. Hospitals without an adequate supply of masks should continue to focus on measures such as extended use, reuse, and reprocessing of their existing supply of masks and respirators. A universal masking policy should always be considered an adjunct to concurrent policies such as visitor restrictions and employee screening for fever and other symptoms of a respiratory illness at their point of entry into the hospital. Similar screening of visitors who are given special exemptions to visit pediatric, obstetric, or hospice patients should also occur daily as they enter the hospital. HCP and exempted visitors who “pass” their daily symptom and signs screen are usually given 1 mask to wear during their entire shift or visit. HCPs are instructed to handle masks only after performing hand hygiene. Masking policies differ slightly across institutions, with some facilities promoting the use of cloth masks versus surgical masks, but the basic premise is the same.

## Challenges with universal masking

There are some theoretical drawbacks to a universal masking policy, the most important of which is the increased cost and depletion of supply of masks in health systems that are already dealing with shortages. Specifically, serious unanticipated supply-chain issues could lead to shortages of masks at a time when the risk of both community and healthcare-associated spread of COVID-19 has increased. Also, logistical issues such as storage of masks during meals or breaks may lead to unanticipated problems such as contamination or loss of masks. Inadvertent self-contamination of masks during a long work shift could theoretically and paradoxically increase the risk of acquisition of SARS-CoV-2. A false sense of security by staff could lead to unintended consequences such as poor hand hygiene or poor adherence to other measures such as social distancing. Compliance with universal masking policies is an additional concern and may in turn lead to time and resource utilization toward compliance monitoring programs or audits.

Published data on the efficacy of universal masking policies to prevent healthcare-associated transmission of respiratory viruses are limited, and the generalizability of these results to the ongoing SARS-CoV-2 pandemic is uncertain. One prospective single-center study that implemented a universal masking policy for all individuals in direct contact with stem cell transplant patients showed a significant reduction in all respiratory viral illnesses on the units where this policy was implemented.^5^ Similar masking policies have been utilized for HCPs who opted out of mandatory influenza vaccination across British Columbia, Canada.^6^ No prospective studies comparing the effectiveness of masking policies during the SARS-CoV-2 pandemic have been undertaken to our knowledge.

## Universal face shields as an alternative

Face shields are face coverings made of clear material attached to a headpiece to cover the eyes, nose, and mouth. This design is intended to protect the facial area and associated mucous membranes from infectious droplets and spatter of body fluids. Face shields have the potential to overcome some of the major drawbacks of face masks. Face shields provide better coverage of the face, compared with masks, thus reducing the risk of self-contamination. Additionally, face shields are durable, and they can be cleaned and reused. Given their simpler design, durability, and reuse potential, face shields are less likely to be in short supply, like face masks. Additionally, face shields do not impede facial nonverbal communication; they can be worn concurrently with other face and eye protective equipment, and they do not impact vocalization. However, lack of a good seal around the face shield may lead to aerosol penetration and may be subject to fogging or glare.^7^ Although additional studies are needed to assess universal face shielding, it offers a promising solution in a time of critical mask shortages.

In conclusion, universal masking when implemented together with strict visitor restrictions and employee screening may incrementally reduce healthcare-associated transmission of SARS-CoV-2. Additionally, such a policy will reduce the burden of contact tracing and subsequent furloughs of HCPs in a time of acute HCP shortages. It also provides reassurance to HCPs as they care for patients with known or suspected COVID-19 infection.

A universal masking policy may not be appropriate for all hospitals because successful implementation of this policy requires an adequate supply of face masks. Furthermore, whether such a policy can indeed prevent transmission of SARS-CoV-2 is uncertain, nor is it known whether the benefits of such a policy outweigh the disadvantages discussed above. Masks are not a substitute for other public health interventions; they must always be used in combination with social distancing and hand hygiene. Future studies are needed to examine the frequency of viral contamination of masks worn for long hours or multiple shifts, as are studies needed to compare rates of healthcare-associated SARS-CoV-2 in hospitals and long-term care facilities that do and do not utilize universal masking policies. Finally, exploring other approaches such as universal use of face shields or more durable face masks could provide much-needed scientific evidence related to the efficacy of universal masking polices or the use of other barrier methods.
